# Correction: Capturing and labeling CO_2_ in a jar: mechanochemical ^17^O-enrichment and ssNMR study of sodium and potassium (bi)carbonate salts

**DOI:** 10.1039/d5sc90243f

**Published:** 2025-11-26

**Authors:** Austin Peach, Nicolas Fabregue, Célia Erre, Thomas-Xavier Métro, David Gajan, Frédéric Mentink-Vigier, Faith Scott, Julien Trébosc, Florian Voron, Nicolas Patris, Christel Gervais, Danielle Laurencin

**Affiliations:** a ICGM, Univ. Montpellier, CNRS, ENSCM Montpellier France austin.peach@umontpellier.fr danielle.laurencin@umontpellier.fr; b CRMN Lyon, UMR 5082 (CNRS, ENS Lyon, Université Lyon 1) Villeurbanne France; c National High Magnetic Field Laboratory (NHMFL), Florida State University Tallahassee Florida USA; d Université de Lille, CNRS, INRAE, Centrale Lille, Université d'Artois FR2638-IMEC-Institut Michel Eugène Chevreul Lille France; e OSU OREME, UAR 3282, Université de Montpellier, CNRS, IRD, INRAE Sète Montpellier France; f HydroSciences Montpellier, UMR 5151, CNRS, IRD, Université de Montpellier Montpellier France; g LCMCP, UMR 7574, Sorbonne Université, CNRS Paris France

## Abstract

Correction for ‘Capturing and labeling CO_2_ in a jar: mechanochemical ^17^O-enrichment and ssNMR study of sodium and potassium (bi)carbonate salts’ by Austin Peach *et al.*, *Chem. Sci.*, 2025, **16**, 10731–10741, https://doi.org/10.1039/D4SC08491H.

The authors regret that after publication they noticed certain errors in the online supplementary information (SI) provided to accompany the article. These errors have been updated in the SI document provided and a list of the changes are given below:

(1) In Table S1, the isolated masses obtained during the synthesis of Na_2_CO_3_·H_2_O were incorrect, as well as the calculated synthetic yields (which are higher than those initially reported). The correct SI table, amended SI text, and a correction to one sentence in the main text of the article are given below.

**Table S1:** Examples of synthesis quantities used in the mechanochemical enrichment of sodium and potassium (bi)carbonate salts

**Table:** 

Product	Water	Mass of hydroxide^*a*^ (mg)	Mass of CDI (mg)	Volume of water (µL)	Product mass (mg)	Synthetic yield^*d*^ (%)
NaHCO_3_	^16^O	45.6^*b*^	368.0	80	76	79
	^17^O	46.2^*b*^	376.0	80	75	77
	^18^O	46.4^*b*^	376.9	80	80	82
Na_2_CO_3_·H_2_O	^16^O	140.7^*c*^	219.3	48	140	84
	^17^O	140.7^*c*^	219.5	48	159	95
	^18^O	140.7^*c*^	219.3	48	151	90
KHCO_3_	^16^O	96.7^*b*^	564.8	125	104	60
	^17^O	93.8^*b*^	541.7	120	119	71
	^18^O	96.7^*b*^	561.8	125	123	71
K_2_CO_3_·1.5H_2_O	^16^O	103.7^*b*^	148.8	35	71	47^*e*^
	^17^O	110.1^*b*^	160.1	35	93	57
	^18^O	93.3^*b*^	135.6	30	78	57

^*a*^Hydroxides used: NaOH or KOH. ^*b*^Educt introduced as pellets. ^*c*^Educt introduced as micropearls. ^*d*^All synthetic yields reported here were calculated taking into consideration the molar mass of non-labeled products, because in practice, the exact enrichment level of each product was not measured experimentally (and hence their exact molar mass is uncertain). Nevertheless, these reported yields were calculated to be to the most 3 to 4% higher than the “real” synthetic yields of the enriched products. ^*e*^The lower synthetic yield for this compound is due to the synthesis being performed on a day with 41% relative humidity, which implies the product was highly deliquescent.

The text under Table S1, which describes the syntheses of Na_2_CO_3_·H_2_O and Na_2_CO_3_, should also be corrected to read:

**Na**_**2**_ **CO**_**3**_ **·H**_**2**_ **O and Na**_**2**_ **CO**_**3**_

H_2_O (2 eq.), NaOH micropearls (2.6 eq.), and CDI (1 eq.) were introduced successively into the milling jar in the following order alongside the stainless steel ball bearings: (i) H_2_O, (ii) two 10 mm balls, (iii) NaOH, and (iv) CDI. The jar was quickly closed, sealed, and the medium milled for 30 minutes at 25 Hz. After milling, the jar was opened and the white paste was scraped using a spatula and transferred on a P4 frit, trying to recover the majority of the product. The rest was recovered with a pipette, by adding absolute EtOH in small fractions to the jar (5 mL in total), which were then added onto the glass frit. The wet precipitate was filtered under dynamic vacuum and washed 3 times with 5 mL of EtOH, followed by 2 × 5 mL Et_2_O. The product was left to dry under vacuum for 1 hour. Once dry, the white solid was scraped from the frit onto aluminum foil and transferred to a vial. Average synthetic yield (*n* = 3): 90 ± 6 %. Typical amount isolated (for quantities in Table S1): *ca.* 150 mg.

To obtain anhydrous Na_2_CO_3_, the monohydrate salt was transferred into a vial, and a heat treatment was performed at 100 °C during *ca.* 3 hours in an oven under air. Average synthetic yield (*n* = 3): 97 ± 2 %. Typical amount isolated (when heat-treating 76 mg of monohydrate): *ca.* 63 mg.

In the main text of the article, the sentence at the end of Section 2.1 should be changed to “Herein, syntheses are described in quantities enabling the isolation of up to *ca.* 150 mg of product (Table S1†).”

(2) Some of the IR spectra in Fig. S5 were plotted on the wrong scale. The corrected Fig. S5 is shown below.


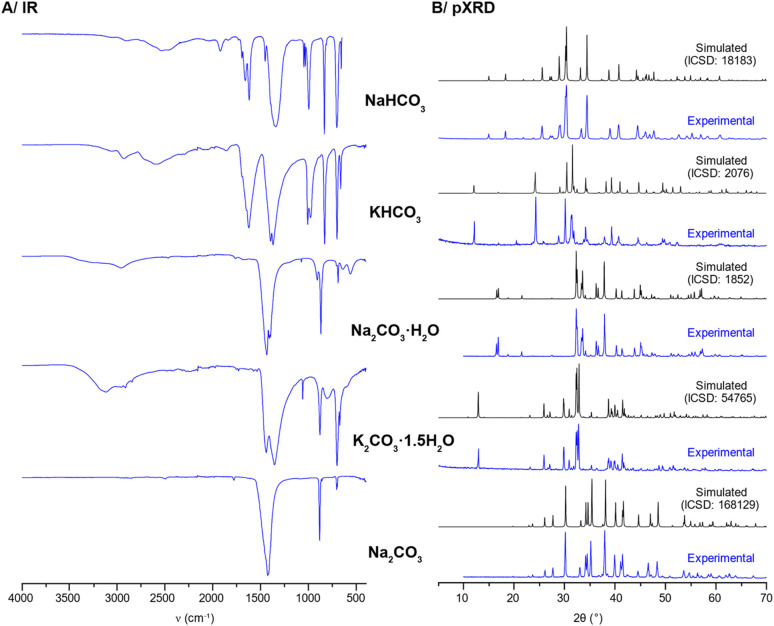
 **Fig. S5** (A) Experimental IR spectra of typical Na and K (bi)carbonate salts obtained after mechanochemical synthesis followed by purification. (B) Experimental pXRD patterns (blue) compared to patterns simulated from reported crystal structures (black) (*λ* ≈ 1.54 Å).

(3) In Table S9, an incorrect ICSD number was given for the structure used in the DFT calculations related to KHCO_3_. The correct ICSD number for the structure of KHCO_3_ used in these calculations is 2076.

(4) In Fig. S12, the analysis of the IR isotopologue vibration-band intensities had been done using the data presented in transmittance mode, while it is the absorbance spectra which are more suitable for peak integrations. Similar conclusions arise here if the IR data used is in absorbance mode, as shown in the corrected version of Fig. S12 and accompanying analysis below.


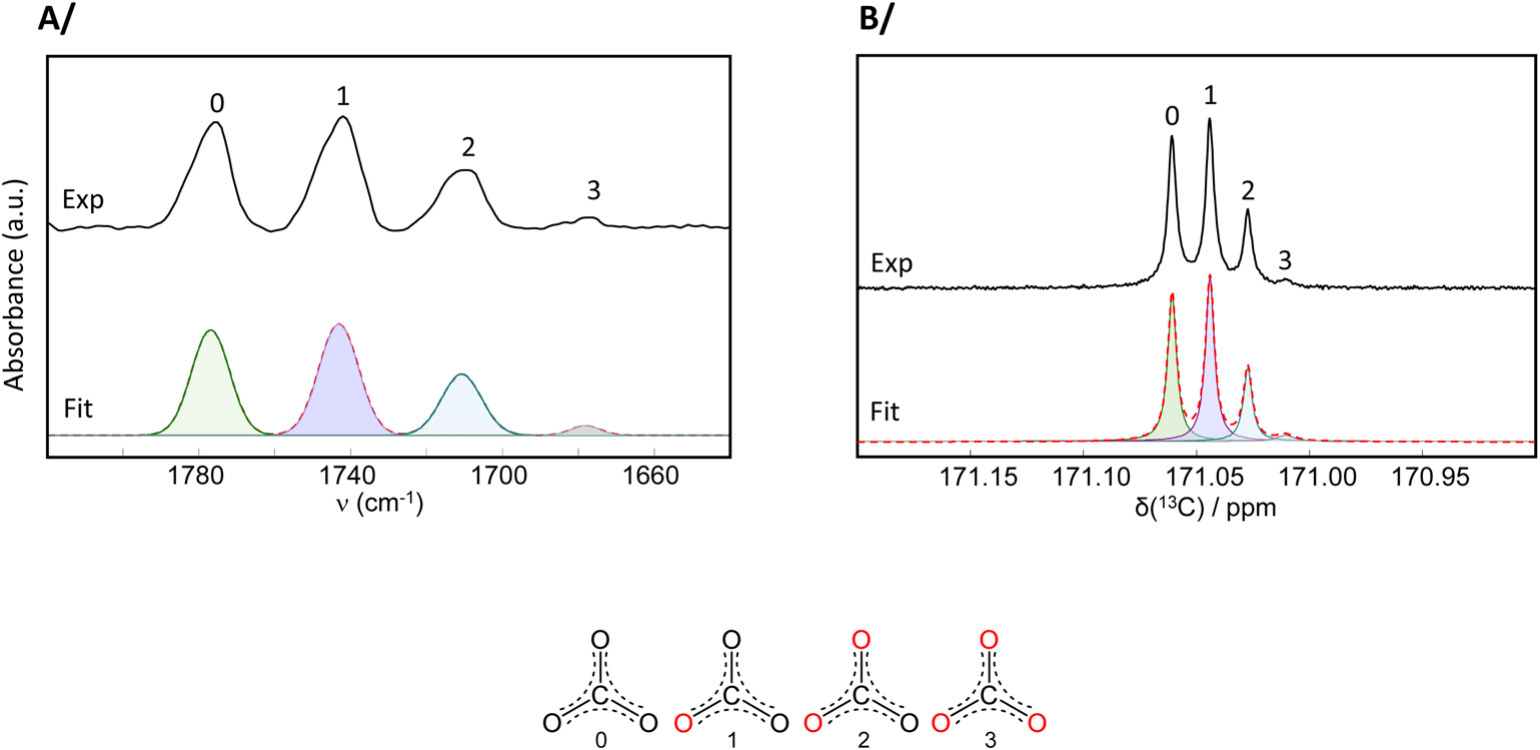
 **Fig. S12** Analyses of an ^18^O-enriched Na_2_CO_3_ phase (prepared using a CDI-based protocol, starting from 99% ^18^O-water), showing the contributions of the different isotopologues: (A) IR spectrum; (B) ^13^C solution NMR spectrum. Experimental spectra are in black, and their overall fits in dashed red (with the contributions from the 4 isotopologues in shaded colours).

By noting %(*I*_*i*_), the relative proportion (in %) of the integral of isotopologue *i* (with *i* = 0, 1, 2, or 3), and considering the ^18^O content of each isotopologue, the average ^18^O-level in the enriched Na_2_CO_3_ was calculated as follows:

From the IR integrals:

%(^18^O) = %(*I*_0_) × 0 + %(*I*_1_) × 0.33 + %(*I*_2_) × 0.66 + %(*I*_3_) × 1

%(^18^O) = 36.07 × 0 + 39.52 × 0.33 + 21.99 × 0.66 + 2.43 × 1

%(^18^O) ≈ 30

From the ^13^C solution NMR integrals:

%(^18^O) = %(*I*_0_) × 0 + %(*I*_1_) × 0.33 + %(*I*_2_) × 0.66 + %(*I*_3_) × 1

%(^18^O) = 37.22 × 0 + 41.50 × 0.33 + 18.47 × 0.66 + 2.81 × 1

%(^18^O) ≈ 29

Overall, based on these analyses, when starting from 99% ^18^O-water, the ^18^O enrichment level achieved in Na_2_CO_3_ is *ca.* 30%.

Hence, by extrapolation, if we assume there are no isotope effects during the labeling procedure used, the following enrichments can be calculated for the ^17^O-labeled products:

*ca.* 27% ^17^O, when starting from 90% ^17^O-labeled water;

*ca.* 21% ^17^O, when starting from 70% ^17^O-labeled water;

*ca.* 12% ^17^O, when starting from 40% ^17^O-labeled water.

(5) In addition, ref. 17 in the SI was given incorrectly. The corrected version of ref. 17 in the SI is:

17. J. O. Thomas, R. Tellgren and I. Olovsson, *Acta Crystallogr. Sect. B Struct. Crystallogr. Cryst. Chem.*, 1974, **30**, 1155–1166.

The Royal Society of Chemistry apologises for these errors and any consequent inconvenience to authors and readers.

